# Multimorbidity and functional decline in community-dwelling adults: a systematic review

**DOI:** 10.1186/s12955-015-0355-9

**Published:** 2015-10-15

**Authors:** Aine Ryan, Emma Wallace, Paul O’Hara, Susan M. Smith

**Affiliations:** HRB Centre for Primary Care Research, Department of General Practice, Royal College of Surgeons in Ireland, 123 St. Stephen’s Green, Dublin 2, Ireland; South East Training Programme for General Practice, General Practice Training Department, Waterford Regional Hospital, Dunmore Road, Waterford, Ireland; Department of Population Health Sciences, Royal College of Surgeons in Ireland, Beaux Lane House, Lower Mercer Street, Dublin 2, Ireland

## Abstract

**Background:**

Multimorbidity affects up to one quarter of primary care populations. It is associated with reduced quality of life, an increased risk of mental health difficulties and increased healthcare utilisation. Functional decline is defined as developing difficulties with activities of daily living and is independently associated with poorer health outcomes. The aim of this systematic review was to examine the association between multimorbidity and functional decline and to what extent multimorbidity predicts future functional decline.

**Methods:**

A systematic literature search (1990-2014) and narrative analysis was conducted. Inclusion criteria: Population; Community-dwelling adults (≥18 years), Risk; Multimorbidity defined as the presence of ≥2 chronic medical conditions in an individual, Primary outcome; Physical functional decline measured using a validated instrument, Study design; cross-sectional or cohort studies. The following databases were included: PubMed, EMBASE, CINAHL, the Cochrane Library and the International Research Community on Multimorbidity (IRCMo) publication list. Methodological quality assessment of included studies was conducted with a suitable risk of bias tool.

**Results:**

A total of 37 studies were eligible for inclusion (28 cross-sectional studies and 9 cohort studies). The majority of cross-sectional studies (*n* = 24/28) demonstrated a consistent association between multimorbidity and functional decline. Twelve of these studies reported that increasing numbers of chronic condition counts were associated with worsening functional decline. Nine cohort studies included 14,133 study participants with follow-up periods ranging from one to six years. The majority (*n* = 5) found that multimorbidity predicted functional decline. Of the five studies that reported the impact of increasing numbers of conditions, all reported greater functional decline with increasing numbers of conditions. One study examined disease severity and found that this also predicted greater functional decline. Overall, cohort studies were of good methodological quality but were mixed in terms of participants, multimorbidity definitions, follow-up duration, and outcome measures.

**Conclusions:**

The available evidence indicates that multimorbidity predicts future functional decline, with greater decline in patients with higher numbers of conditions and greater disease severity. This review highlights the importance of considering physical functioning when designing interventions and systems of care for patients with multimorbidity, particularly for patients with higher numbers of conditions and greater disease severity.

**Electronic supplementary material:**

The online version of this article (doi:10.1186/s12955-015-0355-9) contains supplementary material, which is available to authorized users.

## Background

Multimorbidity is commonly defined as the co-occurrence of two or more chronic medical conditions within an individual [1]. Average life expectancy is rising and so too are the numbers of patients living with multiple chronic medical conditions [2, 3]. One of the main challenges facing both healthcare providers and governments globally is to provide healthcare for the growing numbers of patients living with multiple co-existing diseases [4]. The prevalence of multimorbidity depends on the definition used and the population studied and has been reported from 17–98 % [1, 10–12]. While linked to both deprivation and ageing, this phenomenon is not exclusive to the elderly. In an Australian study 15 % of the 40–59 age group suffered with multiple co-existing medical conditions. A Scottish primary care study demonstrated that the prevalence of multimorbidity increased substantially with age and was present in most of the cohort aged 65 years and older (65–84 years: 64.9 % with multimorbidity). The study also reported that 30.4 % of 45–64 year olds presented with multimorbidity and given the higher numbers of people in this age range, in absolute terms there are more middle aged people with multimorbidity despite the perception that is predominantly an issue for older patients [13]. Multimorbidity is the norm in clinical practice and has been shown to be associated with increased healthcare utilisation, increased emergency hospital admissions and decreased quality of life [14, 15]. It has also been associated with an increased decline in function [16].

Functional decline is defined as a deterioration in self-care skills, where functional autonomy is diminished and disability is increased [5, 6]. A systematic review of 14 cohort studies examining outcomes in older patients admitted to hospital found that functional status predicts length of hospital stay, readmission rates, patient discharge destination and also mortality [7]. In a Japanese longitudinal study of patients over 65 years patients with low Activities of Daily Living (ADL) scores, mortality rate was twice as high over a 5 year follow up compared to patients with higher scores [8]. Functional decline can also lead to increased rates of depression and decreased life satisfaction [6, 9]. Conversely engaging in physical activity is inversely associated with health care utilisation and is associated with increased life satisfaction [6, 9, 17, 18].

It is important for healthcare providers to have a greater understanding of the association between multimorbidity and functional decline considering its impact on patient outcomes. A Cochrane review of interventions to improve outcomes in patients with multimorbidity in primary care suggested that interventions focusing on functional difficulties experienced by patients with multimorbidity may improve outcomes [19]. A previous systematic review completed in 2004 examined the relationship between multimorbidity and quality of life in primary care and reported that multimorbidity is associated with reduced quality of life [20]. However, to date there has been no systematic review of the literature examining the relationship between physical functioning and multimorbidity in community dwelling adults.

The aim of this systematic review was to examine the association between multimorbidity and functional difficulties and whether and to what extent multimorbidity predicts future functional decline.

## Methods

The PRISMA-P Guidelines for reporting systematic reviews were utilised in the conduct of this study [21]. The protocol for this study was published on an international prospective register for systematic reviews (PROSPERO): http://www.crd.york.ac.uk/PROSPERO/display_record.asp?ID=CRD42012003502.

### Data sources

A systematic literature search was carried out using the following search engines: PubMed, EBSCO, EMBASE, CINAHL and the Cochrane library. References from retrieved articles were also searched by hand for relevant articles. The search was carried out from January 1990 to November 2014 and was limited to publications in the English language. Similar systematic reviews have used 1990 as a cut-off date for searches as multimorbidity was a relatively new concept up to that date and does not appear in the literature before this time [16, 19]. Primary healthcare, family practice and family physicians were included as Medical Subject Heading (MeSH) terms. As multimorbidity does not have a MeSH term is was searched for as a keyword and comorbidity was used as a MeSH term.

Two researchers performed the initial screening of titles and abstracts (POH, EW) and irrelevant studies were eliminated. Studies considered eligible for inclusion were read fully in duplicate and their suitability for inclusion to the study was independently determined by two researchers (AR, EW). Any ambiguous findings were discussed with a third researcher (SS) and a consensus reached. Additional information around eligibility was sourced from authors where necessary.

### Inclusion criteria

We included retrospective and prospective cohort studies and cross-sectional study designs. Participants included adults (>18 years) with multimorbidity defined as the presence of two or more chronic medical conditions in an individual [1]. The study setting was primary care or the community. Primary care was defined as: integrated, easy to access health care services by clinicians who are accountable for addressing a large majority of personal health care needs, developing a sustained and continuous relationship with patients, and practicing in the context of family and community [22]. The primary outcome was functional status measured using a validated measure of function e.g. SF-36. Studies carried out in long term care/ residential settings and in-patient settings were excluded. Studies that examined physical function in an index condition and related co-morbidities were also excluded. The current concept of multimorbidity is that no condition is privileged over any other. Studies which investigate index conditions and their co-morbidities also have a different management focus targeting a single disease. This is discussed in a Cochrane review which investigates interventions for patients with multimorbidity [19].

### Assessment of risk of bias

Included studies were assessed for methodological quality using the Cochrane Tool for the Assessment of Bias in Cohort Studies [23] (Additional file 1: Appendix A). This checklist was modified for assessment of the included cross-sectional studies (Additional file 2: Appendix B).

### Statistical analysis

Due to heterogeneity meta-analysis was not possible and a narrative synthesis was conducted. Each article was assessed under the following headings; publication year and country, population and setting, definition and prevalence of multimorbidity, functional decline outcome measure, findings and follow up period (cohort studies).

## Results

**Fig. 1:**
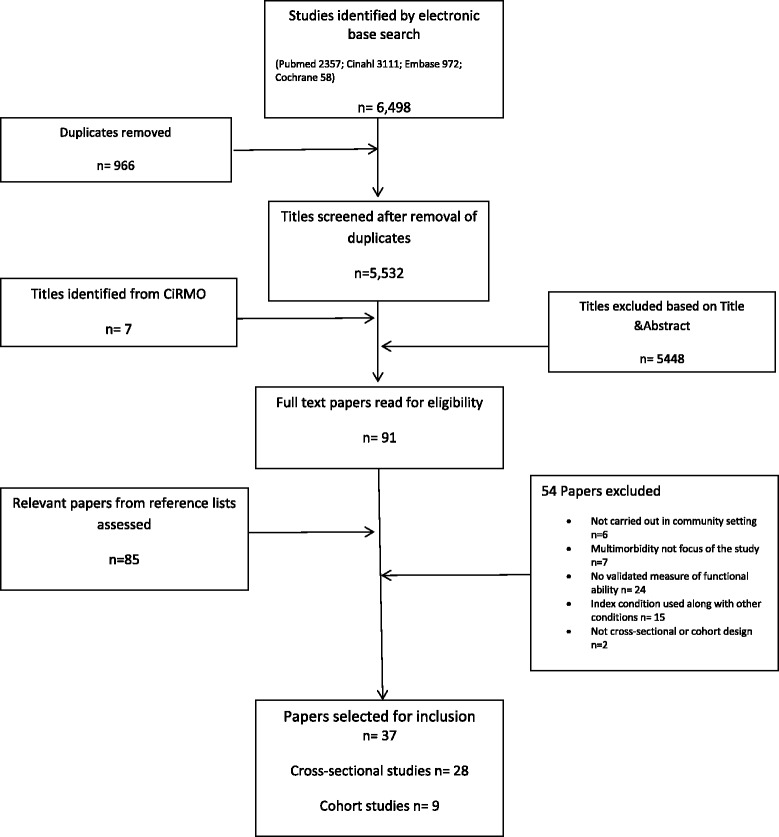
Flow diagram of search

Of 5532 articles screened, 89 were assessed in full text and evaluated according to the study’s inclusion and exclusion criteria. A total of 37 studies were eligible for inclusion: 28 cross-sectional studies [24–52] and 9 cohort studies [53–60]. Figure 1 illustrates the search strategy. Reasons for exclusion of studies are presented in the flow diagram and references provided as an appendix (Additional file 3: Appendix C). Tables 1 and 2 describe the study designs, definitions and outcomes reported in the 37 included studies.

**Table 1:** Included Cross-Sectional Studies

Author Publication Year Country	Population and setting	Definition and prevalence of multimorbidity (MM)	Functional decline outcome measure	Results
Agborsangaya 2012 Canada	Population based survey 4946 adults ≥ 18 years	Self-report chronic conditions from list of 16 19 % with ≥ 2 conditions	EQ–5D	MM associated with a significant reduction in EQ5D index score -0.12
Baker 2000 USA	Primary care clinic 194 adults, 48 % > 60 years	Self-report of > 1 diagnosis according to ICPC categories 51 % > 1 condition	SF– 36	SF36 PCS scores decline with increasing numbers conditions (p < 0.05 on one way MANOVA analysis)
Baker 2006 USA	Community dwelling 247 African American adults, mean age 69	Self-report of doctor-diagnosed conditions used to create condition count Mean 2.9 conditions	AIMS2 (Arthritis Impact Measurement Scale)	No significant association between number of conditions and physical functioning on multivariate analysis
Bayliss M 2012 USA	Population based survey 3877 adults 63 % ≥ 45 years 36 % ≥ 60 years	Self-report of conditions from 26 condition checklist Mean 2.4 conditions Grouped into physical condition MM, mental health MM or both	SF–36	Reduction in PCS score v healthy group: Physical MM : - 6.4 Mental health MM: - 11 Combined physical and mental health MM: -15
Bayliss EA 2012 USA	Primary care, members of HMO 961 aged ≥ 65 years	Self-report of 3 or more conditions from a list of 10 conditions Mean 7.9 conditions	SF–36	Higher condition counts associated with significantly lower PCS scores on multivariate analysis
Brettschneider 2013 Germany	Primary Care – GP databases 3189 patients 65–85 years	Co-existence of 3 or more chronic conditions from a list of 29 Measured by a weighted counts score Mean 7 conditions	EQ–5D	Increased condition count and severity associated with significant reductions in EQ5D-VAS on multivariate analysis
Cesari 2006 Italy	Population – cohort study in mountain community 364 adults ≥ 80 years	Physician report of ≥ 3 conditions based on self-report and chart review 136 patients with ≥ 3 conditions (37.4 %)	Short Physical Performance Battery (SPPB) Four minute walking speed score MDS-HC: ADLs and IADLs	MM significantly associated with lower SPPB score, lower walking speed scores and lower IADLs, but no difference in ADLs
Chen 2011 USA	Population based survey (Behavioural Risk Factor Surveillance System) 430,912 adults ≥18 years	Self-report from list of 8 conditions >90 % participants had ≥ 2 conditions	CDC HRQOL - 4 domains: general health, mental distress, physical distress and activity limitations	Participants with ≥ 3 conditions had highest risk of reporting fair or poor health (AOR 8.7, 95 % CI 8.0 to 9.4)
Cheng 2003 USA	Primary care clinics 316 consecutive clinic attenders 55 –64 years	80 % of patients in the study had ≥ 2 conditions confirmed on chart review	SF–36	Number of conditions significantly associated with decreases in PCS scores in multivariate analysis
Formiga 2005 Spain	Community based 186 adults aged ≥ 90 years	Charlson Comorbidity index, mean score 1.43	Barthel Index (ADLs) Lawton and Brody Scale (IADLS)	Higher comorbidity score significantly associated with worse functional and cognitive capacity on multivariate analysis
Fortin 2007 Canada	Primary care 238 adults attending primary care clinic (randomly collected from a larger study cohort) Mean age 59.0 years	Chart confirmed ≥ two conditions Severity assessed using CIRS score Mean 5.3 (+/-2.8) diagnoses	SF–36	MM measured by simple count was associated with significantly reduced PCS scores on multivariate analysis Increases in MM severity (CIRS scores) associated with greater decreases in PCS scores than simple counts alone
Goins 2010 USA	Community based sample 505 adults ≥ 55 years	Comorbidity scale – self-report from list of 32 conditions Combined with severity assessment using score 0–3 Mean score 5.8	Short Physical Performance Battery (SPPB) Hand grip test	Higher comorbidity scores significantly associated with poor SPPB and hand grip scores in multivariate analysis
Griffith 2010 Canada	Population based sample 8858 adults ≥ 65 years	Self-report of ≥ 2 from list of 12 conditions 72.0 % with ≥ 2 conditions	Multi-dimensional functional assessment questionnaire (OARS)	Combination of foot problems, arthritis and heart problems had most impact on functional disabilities on multivariate analysis A significant association between 11 conditions and functional disability.
Heyworth 2009 UK	Primary care registered patients 4836 adults Mean age 47.9	Self-report from a list of 6 conditions, confirmed by chart review 24 % had ≥ 2 conditions	EQ-5D	Increasing numbers conditions significantly associated with lower EQ5D scores on multivariate analysis
Hunger 2011 Germany	Population based 4412 adults ≥ 65 years	Self-report of ≥ 2 conditions from a list of 6 conditions Prevalence of MM within sample not reported	EQ-5D	Combinations of conditions significantly associated with reduced EQ5D index scores on multivariate analysis (examined in pairs and compared to single condition alone)
Jayasinghe 2009 Australia	Primary care 96 General practices 7606 adults ≥ 18 years Mean age 59.1	Software selected patients with at least one of three chronic conditions. MM: two or more chronic conditions. *n* = 1497 (19.7 %)	SF-12	Number of chronic conditions negatively associated with PCS-12 scores (physical component summary).
Joshi 2003 India	Population based survey 200 adults ≥ 60 years	Self-report of conditions (ICD-10 codes) confirmed by chart review 83 % had ≥ 4 conditions	Standardised Rapid Disability Rating Scale-2	Number of conditions significantly associated with increased mean disability scores
Kadam 2007 UK	Primary care registered patients 9439 aged ≥ 50 years	1. Simple condition counts using chart review for ≥ 2 coded conditions 81 % had ≥ 2 conditions 2. Combinations of 185 selected conditions classified by severity on 4 point scale by GPs	SF-12 dichotomised into poor and good function	Increasing number of conditions significantly associated with poor physical function. AOR 1.6 for 2 or 3 conditions AOR 5 for ≥6 conditions Increasing strength of association between MM severity and poor function
Kadam 2009 UK	Primary care 8791 English aged ≥ 50 years 7753 from Netherlands aged ≥ 18 years	Classified based on 78 conditions which were classified on a 4 point severity index by GPs	SF-12	Higher morbidity severity was significantly associated with poorer physical health on multivariate analysis
Keles 2007 Turkey	Community based survey 4605 parents /grandparents Mean age 53.2 (male) 51.6 (female)	Self-report of conditions from a list of 11 conditions 46 % of participants >1 chronic condition (n ~ 2118)	SF-12	As number of chronic conditions increased physical functioning declined Number of comorbidities an independent predictor of physical functioning
Kim 2012 Korea	Population based survey 1419 adults ≥65 years	Self-report of ≥ 2 conditions from list of 20 conditions Mean 3.88 conditions	EQ-5D	MM significantly associated with lower EQ-5D index score in multivariate analysis
Lawson 2013 UK	Population based survey 7054 aged ≥ 20 years	Self-report of ≥ 2 conditions from list of 40 conditions 18 % ≥ 2 conditions	SF-12 (no breakdown into physical component scores)	Number of conditions all significantly associated with reductions in SF12 scores in multivariate analysis
Michelson 2001 Sweden	Population based survey 3069 adults, Mean age 51	Self-report from list of 13 conditions Categorised into groups - no problems (0 conditions); few problems (1-2 conditions); some problems (3-4 conditions) and a lot (5-13 conditions) 28 % had some or a lot of problems	EORTC QLQ-C30 (HRQOL, specific to Cancer)	Multiple chronic health problems significantly associated with reduced HRQOL adjusted for age
Mujica-Mota 2014 UK	Population based survey 831,537 aged ≥ 18 years	Self-report of ≥ 2 conditions from list of 12 conditions 23 % ≥ 2 conditions	EQ-5D	Number of conditions significantly associated with decrease in EQ-5D scores in multivariate analysis
Noel 2007 USA	Primary care enrolled patients 422 adults Mean age 57 years	≥2 ICD-9 coded conditions from a list of 45 conditions 54 % ≥ 2 conditions	SF-12	Multimorbidity group had significantly lower PCS score (34.8) compared to single morbidity group (39.5)
Parker 2014 UK	Population based survey 5849 adults ≥ 65 years	Self-report of ≥ 2 conditions from list of 15 conditions, verified by chart review HADS score for depression 26 % ≥ 2 conditions	EQ-5D	Total number of conditions not associated with decreased EQ-5D scores on multivariate analysis
Rijken 2005 Netherlands	Primary care sample 1673 chronic disease patients	Coded conditions identified by chart review from list of six conditions 13 % ≥ 2 conditions	SF-36	Multimorbidity associated with significantly lower PCS scores
Wensing 2001 Netherlands	Primary care attenders 4040 adults (28 % ≥ 60 years)	Self-report of ≥ 2 conditions from a list of 25 conditions 16 % ≥ 2 conditions	SF-36	Increasing number conditions associated with lower PCS scores but effect disappeared when controlled for age

**Table 2:** Included Cohort Studies

Author Publication Year Country	Population and setting	Definition and prevalence of multimorbidity (MM)	Functional decline outcome measure/s	Follow-up period Losses to follow-up (%)	Results
Abizanda 2014 Spain	General population (FRADEA Study) 842 adults aged >70 yrs	MM ≥2 chronic diseases in a specific period of time. 14 pre-specified conditions selected for prevalence and impact on disability/mortality Chronic diseases identified from medical records and coded via ICD-10 580 ≥ 2 conditions (69.0 %)	Barthel index (disability) Fried’s criteria (frailty)	2 years 7.5 % loss to follow-up	Disability and frailty was not associated with MM over two years.
Aarts 2012 Netherlands	Primary care (Maastricht Aging Study) 1184 adults aged 21–84 years	MM ≥2 chronic diseases co-occurring within one person Morbidities sourced from GP database including all current and past health problems by clinician 96 included conditions based on medical literature and clinical experience 35.5 % ≥ 2 chronic diseases	SF-36	3 and 6 years 16.4 % loss to follow-up	MM significantly associated with poorer physical functioning at all 3 follow-up points (*p* < 0.001) Significant steep decline in physical function between 3 and 6 year follow up in those with MM (*p* < 0.001) Participants whose morbidity status changed from baseline to 3 year follow up (either to single or MM) associated with significantly lower physical function (*p* < 0.001)
Bayliss 2004 USA	Primary care (Medical Outcomes Study) 2708 adults, mean age 57.6 years	No definition of MM reported Self-report of 7 pre-specified chronic conditions. Condition presence also sourced from records Conditions chosen as of high prevalence in practice and in literature 686 ≥ 2 chronic diseases (25.3 %)	SF-36 (PCS scores)	4 years 41.9 % loss to follow up	≥4 chronic diseases associated with significant decline in physical function (*p* < 0.05) Reduction in PCS by 6.5 used as criteria for clinically significant <4 chronic diseases no association with physical decline Congestive Heart Failure, diabetes and/or respiratory disease predictive of clinically significant decline in PCS (*p* <0.05)
Byles 2005 Australia	Primary care (Veteran’s Affairs Preventative Care Trial) 1417 adults ≥70 years	Co-existence of multiple diseases in the same individual Self-reported MM questionnaire consisting of 25 conditions Severity measure incorporated and included mild cognitive decline 1107 > 3 conditions (78.1 %)	SF-36	2 years 7.2 % lost to follow up	Quality of Life (QoL) decreases as number of conditions increases The presence of each condition associated with significantly lower SF-36 scores (except heart bypass, stroke and diabetes) Data not shown
Drewes 2011 Netherlands	General population (Leiden 85–plus study) 594 adults aged 85 years	MM ≥2 chronic diseases at age 85 years Chart confirmed presence of 9 common conditions pre-specified 234 ≥ 2 chronic diseases (39.4 %)	Groningen Activity Restriction Scale	5 years 53.9 % loss to follow up	Participants with MM had an accelerated progression of ADL (activities of daily living) disability over time compared to those without MM (95 % CI 0.21 -0.63, *p* < 0.001) MM demonstrated accelerated increase in ADL disability in older people with optimal cognitive function (95 % CI 0.39-0.95, *p* < 0.001) This was not observed in participants with lower MMSE scores.
Kiely 1997 USA	Community based (Sample first drawn 1982: Massachusetts state-supported home care programme) 1060 adults aged ≥65 years	No definition of MM reported Self-report of 5 pre-specified medical conditions MM numbers not reported	Functional Dependency Index (FDI)	3 years 22.5 % loss to follow up	Each additional medical condition resulted in a significant increase in the FDI score (*p* < 0.001) Rate of decline did not differ by total number of medical conditions (p =0.67)
Nikolova 2011 Canada	Community based (Research Program on Integrated Services for the Elderly) 1164 disabled adults ≥65 years Disability status estimated using the Functional Autonomy Measurement System (SMAF) Score ≥10 excluded	Comorbidity : number of chronic diseases Self-report of comorbidities using 16 item questionnaire Diseases not specified but grouped into four categories: 0-1 disease 2-3 diseases 4-5 diseases ≥6 diseases 1084 ≥ 2 diseases (93.1 %)	Functional status measured using 7 item IADL subscale of the OAR and Katz ADL index	3 years High rate of attrition discussed but loss to follow up number NR	Comorbidity burden is a strong predictor in developing IADL and ADL disability 6 diseases vs 0–1 disease OR (95 % CI) IADL 6.42 (1.52; 27.18) ADL 16.73 (3.08; 91.06) 4 –5 diseases vs 0–1 disease OR(95 % CI) IADL 1.20 (0.52; 2.80) ADL 0.89 (0.26; 2.98) 2–3 diseases vs 0 –1 disease OR(95 % CI) IADL 1.00 (0.46; 2.20) ADL 1.44 (0.49; 4.15) ≥6 morbidities-6 times more likely to develop ADL disability and 17 times more likely to develop IADL disability
Prior 2011 UK	Primary care 4672 adults aged ≥50 years	Comorbidity –number of chronic diseases Record confirmed condition counts over previous 2 years In addition to number of GP consultations for morbidity in 2 year period Specific cardiovascular and musculoskeletal conditions (*n* = 15) chosen as most prevalent in developed countries Stage of disease as proxy for severity 561 ≥ 1 CVD & MSK condition MM in overall group not reported	SF-12 (PCS)	3 years 46 % loss to follow up	Cardiovascular cohort: higher comorbidity and increasing severity in disease associated with greater deterioration in PCS. Significant deterioration shown for HTN (*p* < 0.001) with PCS score deteriorating by -0.86 over three years Musculoskeletal cohort: no association
Rigler 2002 USA	Community based (Veteran’s Affairs Medical Centre) 492 adults aged ≥65 years	Comorbidity scores: based on sum of the domains affected, and the sum of the domains which patients reported affected function. Self-report of 18 prevalent conditions from 8 organ domains via self-report 335 ≥ 2 diagnoses (68.1 %)	MOS-36 Physical Function Index Self-report ADL and IADL	1 year 7.2 % loss to follow up	Increasing comorbidity significantly associated with increased risk of future functional decline (*p* <0.001) OR 1.09: 2 conditions OR 2.41: ≥ 3 conditions Presence of ADL and IADL problems at baseline demonstrated to have a significant impact on new ADL problems developing at one year (*p* <0.001) OR 4.77: 1 IADL problem at baseline OR 15.6: 1 ADL problem at baseline

### Cross sectional studies

The 28 cross-sectional studies included 1,357,498 participants in total. Overall, 22 of the 28 studies included participants aged over 50 years with 11 of these including participants aged 60 years or older. Sample sizes varied from 186 [33] to 830,537 [47]. The studies were carried out in twelve different countries, the majority in Europe (*n* = 13) and North America (*n* = 11). A total of 22 studies measured multimorbidity using the definition of two or more conditions with three studies using three or more conditions. Two studies used weighted indices such as the Charlson Co-morbidity Index to measure the degree of multimorbidity. Ascertainment of conditions varied between self-report, physician report, chart review and use of software, with the majority (*n* = 17) using self-report to identify conditions. To note, five of the 28 studies did not include mental health conditions [36–39, 50]. There was significant variation in the prevalence of multimorbidity in included studies ranging from 13 % [50] to 90 % [31].

Ten different validated outcome measures were used to measure functional decline in the included 28 studies. Approximately half (46.4 %) used the SF-36 /SF-12, followed by 25 % administering the EQ-5D. The majority of cross-sectional studies demonstrated a consistent association between multimorbidity and functional decline (*n* = 24/28). Twelve of these studies reported that higher condition counts were associated with increased functional decline. In contrast two studies concluded that there was no significant association between the number of conditions and physical functioning [25, 49]. Two studies reported that higher morbidity severity was associated with poorer physical health [34, 42].

### Cohort studies

A total of nine cohort studies included 14,133 study participants with follow-up periods ranging from one to six years [52–60]. Six of these nine studies included participants 65 years or older with sample sizes varying between 492 [60] and 4672 [59]. Four studies were conducted in Europe, four in North America and one study in Australia. The majority defined multimorbidity as ≥2 chronic conditions but most restricted condition inclusion using pre-defined lists ranging from five to 96 conditions. Similar to the cross-sectional studies, self-report was the most prevalent method of ascertaining conditions (*n* = 5), followed by identification through medical record review (*n* = 4). There was similar variation in prevalence rates of multimorbidity in the cohort populations, ranging from 25.3 to 93.1 %. Five different validated outcome measures were used with just over half of the cohort studies (*n* = 5) using the SF-36 or SF-12 as their measure of functional decline.

Seven of the nine cohort studies reported that baseline multimorbidity predicted future functional decline [53–56, 58–60]. Five studies out of nine established that any degree of multimorbidity was predictive of functional decline [53, 55, 56, 59, 60]. Two of these studies stipulated that specific numbers of chronic conditions at baseline were predictive of future decline [54, 58]. The remaining two studies reported that higher numbers of conditions were needed to predict future decline with Nikolova at al. [58] reporting significant functional decline only for those with four or more conditions and Bayliss et al. [17] reporting it for those with six or more conditions. Two studies found no significant relationship between functional disability and multimorbidity over time [52, 57]. Abizanda et al [52] stated that disability and frailty were not associated with multimorbidity over two years [52]. Kiely et al [57] reported that additional conditions were associated with increased impairment at baseline but that functional decline over time did not differ between subjects with no conditions and those with multimorbidity [57].

Five studies examined condition type, disease severity and the impact of cognitive impairment on functional decline. Bayliss et al. examined condition type and reported that those with congestive heart failure, diabetes and/or chronic respiratory disease were at greater risk of functional decline over time compared to other conditions [54]. Prior et al. also examined condition types and reported that those with cardiovascular disease were more likely to have deterioration in physical health compared to those with musculoskeletal conditions [59]. Disease severity was also examined in this study and was found to predict greater functional decline [59]. Two cross-sectional studies examined six different conditions and their combinations [38, 50]. Hunger et al. reported that stroke and bronchitis in combination had the greatest negative impact on function. Rijken et al. stated that combinations of diabetes, cardiovascular disease and chronic respiratory disease lead to a higher risk of physical disability. Drewes et al. examined the role of cognitive impairment in predicting disability in patients with multimorbidity [56]. They found that multimorbidity predicted an accelerated increase in ADL disability in participants with optimal cognitive function at baseline, but not in participants with lower MMSE scores at baseline. This may be explained by the fact that those with poor cognitive function at baseline already had higher levels of disability so had less change in function over time.

One study analyzed the accrual of additional conditions over time and the impact that this had on function decline [53]. The authors reported that participants whose morbidity status changed from baseline to three year follow up (either to single or multimorbidity) had significantly lower physical function at follow up.

#### Risk of bias in included studies

**Fig. 2:**
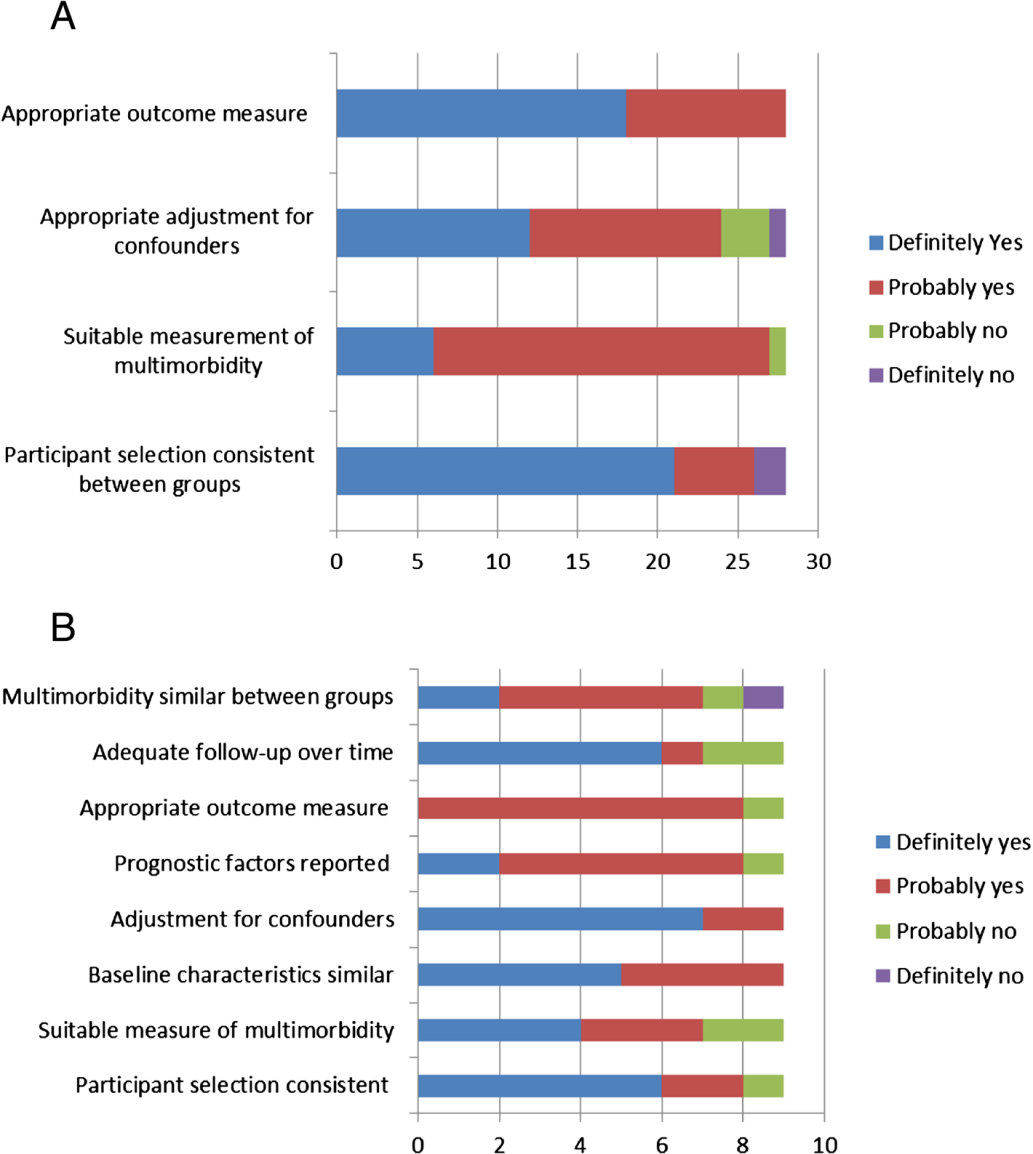
Methodological quality assessment of the included studies as per Cochrane Tool for risk of bias (Additional file 1: Appendix A and Additional file 2: Appendix B). **a** Cross-sectional studies (*n* = 28), **b** Cohort studies (*n* = 9)

Overall, the methodological quality of the included studies was good. The risk of bias assessment is presented in Figs. 2a and b. The majority of cross-sectional studies used valid and reliable outcome measures (*n* = 18) and most studies accounted for possible confounders (*n* = 24). We can also be reasonably confident that multimorbidity was measured appropriately. All papers used valid outcome measures however, blinding and details of assessors was not reported in all. The majority of the cohort studies reported adequate follow up over time (*n* = 6) along with appropriate adjustment for confounding (*n* = 7). Overall, the outcome measures used were suitable and participant groups were well matched.

## Discussion

### Overall findings

This systematic review retrieved 37 relevant studies (nine cohort studies and 28 cross-sectional). Overall the majority of studies demonstrated an association between multimorbidity and functional decline. In addition, 77.8 % (seven out of nine) of the included cohort studies reported that multimorbidity predicts future functional decline. This was more marked with increasing numbers of conditions and was also linked to condition severity. Two cohort studies reported no significant association between functional disability and multimorbidity over time [52, 57]. The conditions included in these studies did not vary considerably from those seven studies which did demonstrate significance (Additional file 4: Appendix D). However, both studies included participants aged 70 years or older [52] and 65 years or older [57]. Abizanda et al. reported that functional ability was impaired at baseline in their participants [52]. It could be argued that the detection of further functional decline was limited due to the age group of participants.

This review adds to the growing evidence base examining the negative impact of multimorbidity on patient outcomes. It highlights a potential cumulative effect in that both multimorbidity and functional decline independently predict poorer outcomes. This review examines one direction of effect, i.e. that baseline multimorbidity predicts future functional decline but it is also possible that poor physical functioning will lead to worsening of multimorbidity, a relationship that our study group plan to examine in an ongoing prospective cohort study in Ireland [61]. For instance, patients with poorer physical function may be less able to engage in physical activity, which may then worsen health through weight gain or other effects on well-being. There is also considerable overlap with the concept of frailty which is also receiving increasing attention in the literature [62].

The findings of this review are consistent with existing evidence linking multimorbidity and poorer health related quality of life [16]. It is also consistent with the qualitative literature exploring the perspectives of patients with multimorbidity, which highlights problems with daily functioning [63]. Some of the impact of multimorbidity on functional decline may relate to the emerging concept of treatment burden [64] as those with multiple conditions are more likely to be attending multiple healthcare providers and undergoing complex treatments.

### Strengths and limitations of this review

We can be reasonably confident in the findings of this review as overall, there was minimal risk of bias in the included studies. However, variation in participants, multimorbidity definitions, follow-up duration, and outcome measures resulted in meta-analysis not being possible. The included studies also varied widely in the number and age of participants. This will have introduced some selection bias for participants. For example, one study reported that 20 % of non-responders and 10 % of responders were less than 40 years [39]. There was also disparity in the prevalence of multimorbidity in the included studies and not all studies examined the impact of numbers of conditions condition type and possible combinations or condition severity. The study settings varied which adds to generalizability though all were conducted in high income countries so results may not apply outside these settings. A further potential limitation was only studies published in English were included.

### Implications of findings

This review highlights the need to carefully consider functional decline in patients with multimorbidity. The Cochrane review of community-based interventions to improve outcomes for people with multimorbidity suggested that such an approach could have a role to play in improving outcomes [19]. Two studies included in the updated Cochrane review had a strong focus on physical functioning and investigated occupational therapist and physiotherapist led interventions [65, 66]. Both studies reported significant improvements in patient outcomes including functional capacity with one demonstrating a reduction in mortality over time [66] Future research should focus on the development and testing of interventions that incorporate a multidisciplinary approach that prioritizes physical function for this patient group. This is particularly important for patients with higher numbers of conditions and greater disease severity. Such an approach was also advocated in a recent clinical management review of multimorbidity, which also suggested that, depending on patient priorities, general practitioners should consider referral to allied health professionals who can intervene to prevent physical decline [67].

Given the complexities highlighted in this review around participant selection and definitions of multimorbidity, future research should be mindful that such variability in terminology exists and carefully consider these issues when developing and reporting interventions [68].

## Conclusion

Multimorbidity is recognised internationally as having a serious impact on health outcomes. This systematic review suggests that multimorbidity predicts future functional decline, which in turn will worsen health outcomes. Interventions are needed that effectively protect physical function in patients with multimorbidity to prevent this inevitable cascade towards poorer health outcomes.

## Additional files

Additional file 1: Appendix A. Tool to Assess Risk of Bias in Cohort Studies (Cochrane). (DOC 43 kb) Additional file 2: Appendix B. Tool to Assess Risk of Bias in Cross-sectional studies. (DOC 39 kb) Additional file 3: Appendix C. Excluded Papers. (DOCX 20 kb) Additional file 4: Appendix D: Included Conditions. (DOC 84 kb)
