# Supplementary material for: Multimorbidity and functional decline in community-dwelling adults: a systematic review
Source: Health Qual Life Outcomes. 2015 Oct 15;13:168. doi: 10.1186/s12955-015-0355-9 (PMC4606907; doi:10.1186/s12955-015-0355-9)
Supplement: Additional file 2: Appendix B. — Tool to Assess Risk of Bias in Cross-sectional studies. (DOC 39 kb) [file 12955_2015_355_MOESM2_ESM.doc]

**Appendix B: Tool to Assess Risk of Bias in Cross-sectional studies**

1. **Was the selection of those with and without Multimorbidity drawn from the same population?**

Definitely yes Probably yes Probably no Definitely no

(low risk of bias) (high risk of bias)

Examples of low risk of bias: Exposed and unexposed drawn for same administrative data base of patients presenting at same points of care over the same time frame.

Examples of high risk of bias: exposed and unexposed presenting to different points of care or over a different time frame.

**2. Can we be confident in the assessment of Multimorbidity?**

Definitely yes Probably yes Probably no Definitely no

(low risk of bias) (high risk of bias)

Examples of low risk of bias: Secure record [e.g. surgical records, pharmacy records]; repeated interview or other ascertainment asking about current use/exposure.

Examples of higher risk of bias: Structured interview at a single point in time; Written self -report; Individuals who are asked to retrospectively confirm their exposure status may be subject to recall bias – less likely to recall an exposure if they have not developed an adverse outcome, and more likely to recall an exposure (whether an exposure occurred or not) if they have developed an adverse outcome.

Examples of high risk of bias: uncertain how exposure information obtained.

**3. Did the study match those with and without Multimorbidity for all variables that are associated with the outcome of interest or did the statistical analysis adjust for these prognostic variables?**

Definitely yes Mostly yes Mostly no Definitely no

(low risk of bias) (high risk of bias)

Examples of low risk of bias: comprehensive matching or adjustment for all plausible prognostic variables.

Examples of higher risk of bias: matching or adjustment for most plausible prognostic variables.

Examples of high risk of bias: matching or adjustment for a minority of plausible prognostic variables, or no matching or adjustment at all. Statements of no differences between groups or that differences were not statistically significant are not sufficient for establishing comparability.

**4. Can we be confident in the assessment of outcome? (Validated measure of functional decline used).**

Definitely yes Probably yes Probably no Definitely no

(low risk of bias) (high risk of bias)

Examples of low risk of bias: Independent blind assessment; Record linkage; For some outcomes (e.g. fractured hip), reference to the medical record is sufficient to satisfy the requirement for confirmation of the fracture.

Examples of higher risk of bias: Independent assessment unblinded; self‐report; For some outcomes (e.g. vertebral fracture where reference to x‐rays would be required) reference to the medical record would not be adequate outcomes.

Examples of high risk of bias: uncertain (no description)
