# Supplementary material for: Multimorbidity and functional decline in community-dwelling adults: a systematic review
Source: Health Qual Life Outcomes. 2015 Oct 15;13:168. doi: 10.1186/s12955-015-0355-9 (PMC4606907; doi:10.1186/s12955-015-0355-9)
Supplement: Additional file 3: Appendix C. — Excluded Papers. (DOCX 20 kb) [file 12955_2015_355_MOESM3_ESM.docx]

**Appendix C –Excluded Papers**

1. Vila-Rodriguez F, Panenka WJ, Lang DJ, Thornton AE, Vertinsky T, Wong H, et al. The hotel study: multimorbidity in a community sample living in marginal housing. The American journal of psychiatry. 2013;170(12):1413-22.
2. Cheville AL, Basford JR, Dos Santos K, Kroenke K. Symptom burden and comorbidities impact the consistency of responses on patient-reported functional outcomes. Archives of physical medicine and rehabilitation. 2014;95(1):79-86.
3. Ganesh S, Guernon A, Chalcraft L, Harton B, Smith B, Louise-Bender Pape T. Medical comorbidities in disorders of consciousness patients and their association with functional outcomes. Archives of physical medicine and rehabilitation. 2013;94(10):1899-907.
4. Bernabeu-Wittel M, Ollero-Baturone M, Ruiz-Cantero A, Moreno-Gaviño L, Barón-Franco B, Fuertes A, et al. Functional Decline Over 1-Year Follow-Up in a Multicenter Cohort of Polypathological Patients: A new Approach to Functional Prognostication. International Journal of Gerontology 6 (2012) 68-74.
5. Xuan J, Kirchdoerfer LJ, Boyer JG, Norwood GJ. Effects of comorbidity on health-related quality-of-life scores: an analysis of clinical trial data. Clinical therapeutics. 1999;21(2):383-403.
6. Grimby A, Svanborg A. Morbidity and health-related quality of life among ambulant elderly citizens. Aging (Milan, Italy). 1997;9(5):356-64.
7. Schlenk EA, Erlen JA, Dunbar-Jacob J, McDowell J, Engberg S, Sereika SM, et al. Health-related quality of life in chronic disorders: a comparison across studies using the MOS SF-36. Quality of life research : an international journal of quality of life aspects of treatment, care and rehabilitation. 1998;7(1):57-65.
8. Idler EL, Russell LB, Davis D. Survival, functional limitations, and self-rated health in the NHANES I Epidemiologic Follow-up Study, 1992. First National Health and Nutrition Examination Survey. American journal of epidemiology. 2000;152(9):874-83.
9. Bower P, Hann M, Rick J, Rowe K, Burt J, Roland M, et al. Multimorbidity and delivery of care for long-term conditions in the English National Health Service: baseline data from a cohort study. Journal of health services research & policy. 2013;18(2 Suppl):29-37.
10. Kempen GI, Ormel J, Brilman EI, Relyveld J. Adaptive responses among Dutch elderly: the impact of eight chronic medical conditions on health-related quality of life. American journal of public health. 1997;87(1):38-44.
11. Tooth L, Hockey R, Byles J, Dobson A. Weighted multimorbidity indexes predicted mortality, health service use, and health-related quality of life in older women. Journal of clinical epidemiology. 2008;61(2):151-9.
12. Patrick DL, Kinne S, Engelberg RA, Pearlman RA. Functional status and perceived quality of life in adults with and without chronic conditions. Journal of clinical epidemiology. 2000;53(8):779-85.
13. Alonso J, Ferrer M, Gandek B, Ware JE, Jr., Aaronson NK, Mosconi P, et al. Health-related quality of life associated with chronic conditions in eight countries: results from the International Quality of Life Assessment (IQOLA) Project. Quality of life research : an international journal of quality of life aspects of treatment, care and rehabilitation. 2004;13(2):283-98.
14. Fried LP, Bandeen-Roche K, Kasper JD, Guralnik JM. Association of comorbidity with disability in older women: the Women's Health and Aging Study. Journal of clinical epidemiology. 1999;52(1):27-37.
15. Marventano S, Ayala A, Gonzalez N, Rodriguez-Blazquez C, Garcia-Gutierrez S, Forjaz MJ. Multimorbidity and functional status in community-dwelling older adults. European journal of internal medicine. 2014;25(7):610-6.
16. Boeckxstaens P, Vaes B, Legrand D, Dalleur O, De Sutter A, Degryse JM. The relationship of multimorbidity with disability and frailty in the oldest patients: a cross-sectional analysis of three measures of multimorbidity in the BELFRAIL cohort. The European journal of general practice. 2015;21(1):39-44.
17. Kriegsman DM, Deeg DJ, Stalman WA. Comorbidity of somatic chronic diseases and decline in physical functioning:; the Longitudinal Aging Study Amsterdam. Journal of clinical epidemiology. 2004;57(1):55-65.
18. Schuz B, Wolff JK, Warner LM, Ziegelmann JP, Wurm S. Multiple illness perceptions in older adults: effects on physical functioning and medication adherence. Psychology & health. 2014;29(4):442-57.
19. Hudon C, Soubhi H, Fortin M. Relationship between multimorbidity and physical activity: secondary analysis from the Quebec health survey. BMC public health. 2008;8:304.
20. Schuz B, Wurm S, Ziegelmann JP, Warner LM, Tesch-Romer C, Schwarzer R. Changes in functional health, changes in medication beliefs, and medication adherence. Health psychology : official journal of the Division of Health Psychology, American Psychological Association. 2011;30(1):31-9.
21. Wolff JL, Starfield B, Anderson G. Prevalence, expenditures, and complications of multiple chronic conditions in the elderly. Archives of internal medicine. 2002;162(20):2269-76.
22. St John PD, Tyas SL, Menec V, Tate R. Multimorbidity, disability, and mortality in community-dwelling older adults. Canadian family physician Medecin de famille canadien. 2014;60(5):e272-80.
23. McLean G, Gunn J, Wyke S, Guthrie B, Watt GC, Blane DN, et al. The influence of socioeconomic deprivation on multimorbidity at different ages: a cross-sectional study. The British journal of general practice : the journal of the Royal College of General Practitioners. 2014;64(624):e440-7.
24. Van Den Bos 1995 Van Den Bos, Geertrudis AM. "The burden of chronic diseases in terms of disability, use of health care and healthy life expectancies." *The European Journal of Public Health* 5.1 (1995): 29-34.
25. Barros MB, Zanchetta LM, Moura EC, Malta DC. Self-rated health and associated factors, Brazil, 2006. Revista de saude publica. 2009;43 Suppl 2:27-37.
26. Duguay C, Gallagher F, Fortin M. The experience of adults with multimorbidity: a qualitative study. Journal of Comorbidity 2014; 4: 11-21.
27. Dy SM, Pfoh ER, Salive ME, Boyd CM. Health-related quality of life and functional status quality indicators for older persons with multiple chronic conditions. Journal of the American Geriatrics Society. 2013;61(12):2120-7.
28. Slater M, Perruccio AV, Badley EM. Musculoskeletal comorbidities in cardiovascular disease, diabetes and respiratory disease: the impact on activity limitations; a representative population-based study. BMC public health. 2011;11:77.
29. Fuchs Z, Blumstein T, Novikov I, Walter-Ginzburg A, Lyanders M, Gindin J, et al. Morbidity, comorbidity, and their association with disability among community-dwelling oldest-old in Israel. The journals of gerontology Series A, Biological sciences and medical sciences. 1998;53(6):M447-55.
30. Beland F, Zunzunegui MV. Predictors of functional status in older people living at home. Age and ageing. 1999;28(2):153-9.
31. Chapleski EE, Lichtenberg PA, Dwyer JW, Youngblade LM, Tsai PF. Morbidity and comorbidity among Great Lakes American Indians: predictors of functional ability. The Gerontologist. 1997;37(5):588-97
32. Miller RR, Zhang Y, Silliman RA, Hayes MK, Leveille SG, Murabito JM, et al. Effect of medical conditions on improvement in self-reported and observed functional performance of elders. Journal of the American Geriatrics Society. 2004;52(2):217-23.
33. Marengoni A, von Strauss E, Rizzuto D, Winblad B, Fratiglioni L. The impact of chronic multimorbidity and disability on functional decline and survival in elderly persons. A community-based, longitudinal study. Journal of internal medicine. 2009;265(2):288-95.
34. Nutzel A, Dahlhaus A, Fuchs A, Gensichen J, Konig HH, Riedel-Heller S, et al. Self-rated health in multimorbid older general practice patients: a cross-sectional study in Germany. BMC family practice. 2014;15:1.
35. Perruccio AV, Power JD, Badley EM. The relative impact of 13 chronic conditions across three different outcomes. Journal of epidemiology and community health. 2007;61(12):1056-61.
36. Mor V, Wilcox V, Rakowski W, Hiris J. Functional transitions among the elderly: patterns, predictors, and related hospital use. American journal of public health. 1994;84(8):1274-80.
37. Guralnik JM, LaCroix AZ, Abbott RD, Berkman LF, Satterfield S, Evans DA, et al. Maintaining mobility in late life. I. Demographic characteristics and chronic conditions. American journal of epidemiology. 1993;137(8):845-57.
38. Zeng C, Ellis JL, Steiner JF, Shoup JA, McQuillan DB, Bayliss EA. Assessment of morbidity over time in predicting health outcomes. Medical care. 2014;52 Suppl 3:S52-9.
39. Vogeli C, Shields AE, Lee TA, Gibson TB, Marder WD, Weiss KB, et al. Multiple chronic conditions: prevalence, health consequences, and implications for quality, care management, and costs. Journal of general internal medicine. 2007;22 Suppl 3:391-5.
40. Mond JM, Baune BT. Overweight, medical comorbidity and health-related quality of life in a community sample of women and men. Obesity (Silver Spring, Md). 2009;17(8):1627-34.
41. Sikorski C, Luppa M, Weyerer S, Konig HH, Maier W, Schon G, et al. Obesity and associated lifestyle in a large sample of multi-morbid German primary care attendees. PloS one. 2014;9(7):e102587.
42. Fowler-Brown A, Wee CC, Marcantonio E, Ngo L, Leveille S. The mediating effect of chronic pain on the relationship between obesity and physical function and disability in older adults. Journal of the American Geriatrics Society. 2013;61(12):2079-86.
43. Baune BT, Adrian I, Jacobi F. Medical disorders affect health outcome and general functioning depending on comorbid major depression in the general population. Journal of psychosomatic research. 2007;62(2):109-18.
44. Yeo J, Karimova G, Bansal S. Co-morbidity in older patients with COPD--its impact on health service utilisation and quality of life, a community study. Age and ageing. 2006;35(1):33-7.
45. Noel PH, Williams JW, Jr., Unutzer J, Worchel J, Lee S, Cornell J, et al. Depression and comorbid illness in elderly primary care patients: impact on multiple domains of health status and well-being. Annals of family medicine. 2004;2(6):555-62.
46. Aydemir O, Ozdemir C, Koroglu E. The impact of co-morbid conditions on the SF-36: a primary-care-based study among hypertensives. Archives of medical research. 2005;36(2):136-41.
47. Wijlhuizen GJ, Perenboom RJ, Garre FG, Heerkens YF, van Meeteren N. Impact of multimorbidity on functioning: evaluating the ICF Core Set approach in an empirical study of people with rheumatic diseases. Journal of rehabilitation medicine. 2012;44(8):664-8.
48. Wee HL, Cheung YB, Li SC, Fong KY, Thumboo J. The impact of diabetes mellitus and other chronic medical conditions on health-related Quality of Life: is the whole greater than the sum of its parts? Health and quality of life outcomes. 2005;3:2.
49. van Manen JG, Bindels PJ, Dekker EW, Ijzermans CJ, Bottema BJ, van der Zee JS, et al. Added value of co-morbidity in predicting health-related quality of life in COPD patients. Respiratory medicine. 2001;95(6):496-504.
50. Loza E, Jover JA, Rodriguez L, Carmona L. Multimorbidity: prevalence, effect on quality of life and daily functioning, and variation of this effect when one condition is a rheumatic disease. Seminars in arthritis and rheumatism. 2009;38(4):312-9.
51. Fossa SD, Hess SL, Dahl AA, Hjermstad MJ, Veenstra M. Stability of health-related quality of life in the Norwegian general population and impact of chronic morbidity in individuals with and without a cancer diagnosis. Acta oncologica (Stockholm, Sweden). 2007;46(4):452-61.
52. Erickson SR, Christian RD, Jr., Kirking DM, Halman LJ. Relationship between patient and disease characteristics, and health-related quality of life in adults with asthma. Respiratory medicine. 2002;96(6):450-60.
53. Egede LE. Diabetes, major depression, and functional disability among U.S. adults. Diabetes care. 2004;27(2):421-8.
54. Kadam UT, Croft PR. Clinical comorbidity in osteoarthritis: associations with physical function in older patients in family practice. The Journal of rheumatology. 2007;34(9):1899-904.
