# Supplementary material for: Multimorbidity and functional decline in community-dwelling adults: a systematic review
Source: Health Qual Life Outcomes. 2015 Oct 15;13:168. doi: 10.1186/s12955-015-0355-9 (PMC4606907; doi:10.1186/s12955-015-0355-9)
Supplement: Additional file 4: Appendix D: — Included Conditions. (DOC 84 kb) [file 12955_2015_355_MOESM4_ESM.doc]

**Appendix D: Included Conditions**

Cohort Studies

| **Author**  **Publication**  **Year**  **Country** | **No of conditions** | **Included conditions** |
| --- | --- | --- |
| Abizanda  2014  Spain | 14 conditions | Hypertension, dislipemia, diabetes, depression, coronary disease, atrial fibrillation, COPD  dementia, asthma /bronchial hyperactivity, stroke, non-skin cancer, heart failure, anaemia  Parkinsonism |
| Aarts  2012 Netherlands | 96 conditions | Malignancies, movement disorders, chronic respiratory diseases, cardiovascular diseases, endocrine diseases, neurological disturbances, psychological disorders  Conditions interfering with cognition excluded:  History of coma, cerebrovascular disorder, tumour of the nervous system, MS, Parkinsonism, epilepsy, dementia, organic psychosis, schizophrenia, affective psychosis, mental retardation  congenital malformation |
| Bayliss  2004 USA | 7 conditions | Hypertension, congestive heart failure , myocardial infarction (within past 6/12), diabetes, depression, musculoskeletal disease, respiratory disease |
| Byles  2005 Australia | 25 conditions | Arthritis rheumatism, vison problems, blood pressure, sciatica, back or spinal problems, hearing problems, forgetfulness, digestion problems, urinary tract problems, chronic allergic or sinus problems, dermatitis or other skin condition, chronic pain, angina or chest pain, depression, cancer, chronic lung disease, muscle weakness or spasm, heart attack, gall bladder trouble, fits/ faints/ funny turns, heart bypass, kidney problems, diabetes , stroke, heart failure , liver problems |
| Drewes  2011 Netherlands | 9 conditions | Arthritis, depressive symptoms , history of Cancer, diabetes mellitus, heart failure, COPD, myocardial infarction, stroke, Parkinson disease |
| Kiely  1997  USA | Self-report of 5 pre-specified medical conditions | Heart problem, arthritis, diabetes, cancer, stroke |
| Nikolova  2011 Canada | 1084 > 2 diseases (93.1%) | Diseases not specified but grouped into four categories:   - 1. disease   2-3 diseases  4-5 diseases  ≥6 diseases |
| Prior  2011  UK | Specific cardiovascular and musculoskeletal conditions (n=15) chosen as most prevalent in developed countries | CVD: hypertension, atrial fibrillation, ischaemic heart disease, angina, myocardial infarction, heart failure  MSK: soft tissue disorder, soft tissue pain, peripheral enthesopathies, joint disorders, back pain, neck pain, osteoarthritis, osteoporosis, inflammatory polyarthropathy |
| Rigler  2002 USA | 18 prevalent conditions from 8 organ domains via self-report | Cardiovascular: angina, congestive heart failure, heart attack  Respiratory: lung disease (emphysema, asthma, bronchitis)  Musculoskeletal: osteoporosis, broken bone, joint replacement, joint fusion, amputation  Neurologic: Parkinson’s disease, stroke  General: insomnia, chronic pain, anxiety/ depression, cancer, diabetes mellitus  Visual: glaucoma, cataract |

Cross-Sectional Studies

| Author  Publication  Year  Country | No of conditions | Included conditions |
| --- | --- | --- |
|  |  |  |
| Agborsangaya2012  Canada | 16 conditions | Diabetes, chronic obstructive pulmonary disorder, asthma, high blood pressure, high cholesterol, sleep apnea, congestive heart failure, obesity, depression or anxiety, chronic pain, arthritis, heart disease, stroke (or related), cancer, kidney disease, GIT disease. |
| Baker  2000 USA | Number not detailed  Four ICPC categories included | 1. Circulatory: HTN, coronary artery disease, myocardial infarction, angina, stroke, congestive heart failure, other circulatory 2. Endocrine: diabetes 3. Respiratory: asthma, emphysema and chronic obstructive pulmonary disease 4. Musculoskeletal: low back pain, neck /upper limb pain, osteoarthritis, degenerative joint disease, other musculoskeletal pain, other musculoskeletal problems. |
| Baker  2006 USA | Number not detailed | Conditions included not detailed in paper |
| Bayliss M  2012 USA | 26 conditions | Cardiovascular cluster: heart attack in past year, congestive heart failure, angina, coronary artery disease, other heart conditions.  Gastrointestinal cluster: liver disease, kidney disease, stomach disease, irritable bowel syndrome  Endocrine cluster: type 1, type 2 diabetes  Musculoskeletal cluster: rheumatoid arthritis, osteoarthritis, degenerative arthritis, osteoporosis  Respiratory cluster: chronic obstructive pulmonary disease |
| Bayliss EA  2012 USA | 10 conditions | Conditions included not detailed in paper |
| Brettschneider  2013  Germany | 45 conditions | Depression, hypertension, thyroid dysfunction, obesity, chronic low back pain, neuropathies, anaemias, asthma/ COPD, cardiac insufficiency, osteoporosis, chronic stroke, joint arthrosis, lipid metabolism disorders, Parkinson’s disease, urinary incontinence, anxiety disorders, atherosclerosis, cardiac valve disorders, diabetes mellitus, insomnia, renal insufficiency, severe hearing loss, coronary heart disease, dizziness, intestinal diverticulosis, psoriasis, rheumatism, urinary tract calculi, lower limb varicosis, allergies, chronic ischaemic heart disease, cardiac arrhythmias, severe vison reduction, cancers, gout, intestinal diverticulosis, chronic gastritis, prostatic hyperplasia, cardiac valve disorders, chronic cholecystitis, liver diseases, somatoform disorders, obesity, migraine/ chronic headache, non-inflammatory gynaecological problems |
| Cesari  2006 Italy | 14 conditions | Obesity, coronary heart disease, cerebrovascular disease, congestive heart failure, peripheral heart disease, hypertension, lung disease, osteoarthritis, diabetes, dementia, Parkinson’s disease, renal failure, cancer, depression |
| Chen  2011  USA | 8 conditions | Asthma, arthritis, myocardial infarction, angina, stroke, diabetes, obesity, hypertension |
| Cheng  2003 USA | 7 conditions | Hypertension, arthritis, diabetes type 2, heart disease, pulmonary disease, visual and hearing impairment, osteoporosis |
| Formiga  2005 Spain | 7 conditions | Hypertension, diabetes mellitus, dyslipidemia, previous stroke, ischaemic heart disease, chronic obstructive lung disease, heart failure |
| Fortin  2007 Canada | Conditions not detailed  14 CIRS organ domains outlined | Cardiac, vascular, haematological, respiratory, ophthalmologic, upper gastrointestinal, lower gastrointestinal, hepatic and pancreatic, renal, genitourinary, musculoskeletal, neurological, endocrine metabolic and breast, psychiatric |
| Goins  2010 USA | 32 conditions | Angina, congestive heart failure, heart attack, lung disease, arthritis, osteoporosis, broken bone, joint replacement, joint fusion, amputation, Parkinson’s disease, stroke, depression/ anxiety, sleep problem, back pain, chronic pain syndrome, cancer, diabetes, glaucoma, cataracts, heart disease, high blood pressure, ulcer or stomach disease, kidney disease, liver disease, anaemia or other blood disease, vision loss, hearing loss, skin disorders, epilepsy, multiple sclerosis, urinary tract disorders |
| Griffith  2010 Canada | 12 conditions | Cognitive impairment, Parkinson’s disease, hypertension, heart problems, stroke, diabetes, respiratory problems, hearing problems, vison problems, arthritis, foot problems, fracture |
| Heyworth  2009 UK | 6 conditions | Asthma, COPD, ischaemic heart disease, hypertensive disease, diabetes mellitus, cerebrovascular disease |
| Hunger  2011 Germany | 6 conditions | Stroke, coronary disorders, cancer, diabetes, hypertension, chronic bronchitis |
| Jayasinghe  2009  Australia | 3 conditions. | Asthma, type 2 diabetes, hypertension/ischaemic heart disease |
| Joshi  2003 India | 27 conditions | Anaemia, dental problems, hypertension, chronic obstructive airway disease, cataract, osteoarthritis, skin and nail infections, urinary incontinence, senile pruritus, senile deafness, paraesthesia, prostate enlargement, valvular heart disease, acid peptic disease, cervical spondylosis, anxiety neurosis, corneal ulcer, asthma, diabetes mellitus, obesity, conjunctivitis, rheumatoid arthritis, sciatica, psychosis, pulmonary tuberculosis, haemorrhoids, faecal incontinence |
| Kadam  2007  UK | 19 domains according to Read system | Conditions included not detailed in paper |
| Kadam  2009  UK | 78 conditions | Asthma, anxiety, allergic rhinitis, high blood pressure, osteoarthritis, diabetes mellitus, hypercholesterolemia, hypothyroidism, emphysema, heart disease, obesity, rheumatoid arthritis, haemorrhoids  Not all included conditions detailed in paper |
| Keles  2007  Turkey | 11 conditions | Asthma, chronic bronchitis, heart disease, hypertension, peptic or duodenal ulcer, diabetes mellitus, joint diseases, liver/ kidney disease, stroke, cancer, other chronic disease |
| Kim  2012 Korea | 20 conditions | Hypertension, chronic kidney disease, obesity, diabetes mellitus, hypercholesterolemia, anaemia, ischaemic heart disease, stroke, respiratory disease, back pain, depression, urinary incontinence, thyroid disease, cataract and glaucoma, cancer, peptic ulcer, chronic liver disease, arthritis, osteoporosis, pulmonary tuberculosis |
| Lawson  2013  UK | 40 conditions | Cancer, diabetes, other endocrine/metabolic, mental illness, mental handicap, epilepsy, other nervous system problems, cataract, other eye complaints, poor hearing/deafness, tinnitus, Meniere’s disease, other ear complaints, stroke, heart attack/angina, hypertension, other heart problems, haemorrhoids, varicose veins, other blood vessel problems, bronchitis, asthma, hay fever, other respiratory complaints, stomach ulcer, other digestive complaints, complaints of bowel, complaints of teeth/mouth/tongue, kidney complaints, urinary tract infection, other bladder problems, reproductive system disorders, arthritis, back problems, other bone/joint/muscle problems, infectious disease, disorder of blood, skin complaints, other complaints |
| Michelson  2001 Sweden | 13 conditions | Cardiac infarction, cancer, diabetes, angina, asthma, skin problems, allergies, long-standing medication, arthritis, reduced limb function, blood pressure, hearing impairment, visual impairment |
| Mujica-Mota  2014  UK | 12 conditions | Angina/heart, arthritis/joint, asthma, cancer, deaf/severe hearing, diabetes, epilepsy, high blood pressure, kidney/liver disease, back problems, mental health, neurological |
| Noel  2007 USA | 45 conditions | Hypertension, hyperlipidemia, diabetes, ischaemic heart disease, obesity, osteoarthritis, low back pain, GERD, benign prostatic hyperplasia, depression, PTSD, other anxiety disorder, alcohol abuse, substance abuse, bipolar disorder, schizophrenia  Not all included conditions detailed in paper |
| Parker  2014  UK | 15 conditions | Cardiac arrhythmia, cancer, cerebrovascular disease, stroke /transient ischaemic attack, dementia, diabetes, heart valve and valvular disease, hypertension, ischaemic coronary heart disease, peripheral vascular disease, pulmonary disease, renal disease, rheumatoid arthritis, osteoarthritis, neurological disease |
| Rijken  2005 Netherlands | 6 conditions | Cardiovascular disease, cancer, arthritis, chronic respiratory disease, diabetes mellitus, thyroid dysfunction |
| Wensing  2001 Netherlands | 25 conditions | Asthma/ chronic bronchitis/ COPD, infection of nose/sinus/throat, severe heart disease /heart infarction, high blood pressure, consequences of stroke, stomach/bowel ulcer, severe bowel problems, gall stones/ gall bladder infection, liver disease/liver cirrhosis, kidney stones, severe kidney disease, chronic bladder infection, female prolapse, diabetes mellitus, thyroid disease, chronic back pain, arthrosis of knees/hips/hands, infection of joint in hands/ feet, other chronic reuma, epilepsy, dizziness/falling, migraine, severe skin disease, malignant disease/ cancer, other disease/ health problem |
